# The Fibrotic–Cancer Continuum in IPF: Shared Mechanisms, Clinical Implications and Therapeutic Challenges

**DOI:** 10.3390/life16020295

**Published:** 2026-02-09

**Authors:** Panagiota Tsiri, Marousa Kouvela, Ourania Papaioannou, Vasilina Sotiropoulou, Matthaios Katsaras, Nikolaos Syrigos, Fotios Sampsonas, Argyrios Tzouvelekis

**Affiliations:** 1 Department of Internal and Respiratory Medicine, Medical School, University of Patras, 26504 Patras, Greece; tsiripanayiota@gmail.com (P.T.); ouraniapapaioannou@hotmail.com (O.P.); matthewkat1@gmail.com (M.K.);; 2 Oncology Unit, Third Department of Internal Medicine, Sotiria General Hospital for Chest Diseases, National and Kapodistrian University of Athens, 11527 Athens, Greece; markouvela@yahoo.gr (M.K.); nksyrigos@gmail.com (N.S.); 3 Department of Pulmonary, Critical Care and Sleep Medicine, Yale School of Medicine, New Haven, CT 06511, USA

**Keywords:** idiopathic pulmonary fibrosis, lung cancer, pathogenesis, fibrosis–cancer continuum, therapeutic targets

## Abstract

Idiopathic pulmonary fibrosis represents a chronic, progressive, lethal lung disease of various etiologies exerting a dramatic impact on patients’ survival and quality of life. Its increasing prevalence and high mortality rates indicate the importance of early diagnosis and management involving the assessment of specific comorbidities, such as lung cancer. Emerging evidence suggests that in the context of IPF, lung scarring may be a potential risk factor for lung cancer development. Both disease entities present pathogenic commonalities including genetic and epigenetic markers, signaling pathways and cell transformation obtaining mesenchymal phenotypes. Beyond understanding disease pathogenesis, anti-cancer drugs such as nintedanib have been successfully used to treat patients with IPF. Additionally, a therapeutic approach that includes a mix of various pleiotropic anti-fibrotic agents is currently being developed for IPF treatment. Currently, there is no consensus on the application of therapeutic algorithms in concurrent pulmonary fibrosis and lung tumors. This review summarizes the current state of knowledge on common cellular and molecular pathogenetic mechanisms of IPF and lung cancer and highlights potential therapeutic targets with fruitful results.

## 1. Introduction

Idiopathic pulmonary fibrosis (IPF) is a chronic, lethal interstitial lung disease that represents an important health burden and an imperative therapeutic need. It predominantly affects elderly patients, with an overall median survival of 3 to 5 years following diagnosis, if left untreated [1]. Although the etiology of IPF remains largely unknown, current evidence indicates that it represents an abnormal wounding response to repetitive local epithelial microinjuries resulting in cell apoptosis, denudation of epithelial cell membrane, excessive deposition of extracellular matrix, differentiation of fibroblasts into myofibroblasts, and areas of dense fibrosis and honeycombing, leading ultimately to lung function decline, respiratory failure and death [2]. Besides environmental factors such as smoking, viral infectious diseases, and occupational exposures, genetic abnormalities ranging from common polymorphisms to rare familial subtypes are involved in lung fibrogenesis [3].

Despite the emergence of two anti-fibrotic compounds, pirfenidone and nintedanib, the prognosis of IPF remains discouraging as both drugs seem only to slow down disease progression, and thus leaving patients with major functional disability [4]. Importantly, nearly 25% of patients with IPF die from non-IPF related causes including major comorbidities such as cardiovascular (8–10%) and lung cancer (10%). The latter exerts a dramatic impact on disease course and patients’ quality of life and survival. On the other hand, pulmonary fibrosis represents a major risk factor for lung carcinogenesis, denominated “scarcinoma”. Based on the recent US Preventive Services Task Force Recommendation Statement, patients with pulmonary fibrosis appear in high-risk groups for lung cancer development [5,6]. According to large epidemiological studies, the prevalence of lung cancer in pulmonary fibrosis patients ranges from 4.4% to 13% [7,8]. Of note, the cumulative incidence of lung cancer increases over time, being 3.3% one year after diagnosis and 54.7% after ten years in survivors with lung fibrosis [9,10,11,12]. Even patients with asymptomatic interstitial lung abnormalities present with an increased risk of cancer in the National Lung Screening Trial (incidence rate ratio 1.33; 95% CI: 1.07–1.65) [13]. Incidence of lung cancer is also increased in non-IPF fibrosis, suggesting a role for inflammation and fibrosis in the development of lung tumors [14]. Besides tobacco smoking [15], IPF and lung cancer share striking pathogenic commonalities including microsatellite instability, epigenetic alterations, telomere attrition and impaired cellular bioenergetics [4,16,17,18,19]. Recent molecular studies identified potentially actionable alterations in different genes in various histologic subtypes [20]. A growing scientific interest in the intersection between pulmonary fibrosis and lung cancer has been documented in a recent bibliometric analysis covering research activity from 2004 to 2024. This study demonstrated a marked increase in publications addressing the coexistence of fibrosis and malignancy, identifying major thematic clusters related to epidemiology, mechanistic pathways, molecular profiling, and emerging therapeutic strategies, thereby underscoring the expanding global relevance of the IPF–lung cancer continuum [21].

Despite abundant mechanistic links between pulmonary fibrosis and lung cancer, there is a considerable lack of knowledge on the diagnostic and therapeutic management of patients diagnosed with both clinical entities. Past (ATS/ERS 2011) and current ATS/ERS/JRS/ALAT guidelines (2022) for IPF do not address this crucial issue [22,23]. This has been clearly shown by an international survey, called DIAMORFOSIS (DIAgnosis and Management Of lung canceR and FibrOSIS) in which 28% of participants reported lack of awareness for the coexistence of these two entities while only 17% (2/12) of posed questions reached a consensus (>70% agreement) among 494 expert physicians across the globe [24]. The recent Japanese guideline on the management of IPF underlines the impact of comorbid lung cancer on patients’ survival and quality of life and the need for large randomized controlled trials enrolling patients with fibrotic ILD and lung cancer [25]. Importantly, the identification of a solitary nodule on HRCT in patients with IPF represents a major pitfall for practicing clinicians due to challenging diagnostic approaches. Interventions including procedures such as bronchoscopy, CT-guided transthoracic needle biopsy or surgery are quite often limited by the patients’ performance status and the presence of comorbidities such as emphysema, and could be proven detrimental for ILD progression. Currently, diagnostic and therapeutic approaches to lung cancer in IPF are a matter of debate since guideline-based [26,27], stage-appropriate approaches to treat lung cancer may be associated with a significant progression of the fibrotic ILD or significant complications such as acute exacerbations, infections or immune-related pneumonitis, which again are associated with high morbidity and mortality [9]. It is important to underline that epidemiological and therapeutic data in the field of fibrotic ILD and lung cancer are predominantly from Asian countries and there is major lack of evidence at a pan-European or North American level. In some countries, patients with fibrotic ILD and concomitant lung cancer are occasionally excluded from the current anti-fibrotic standard of care.

**Figure 1:**
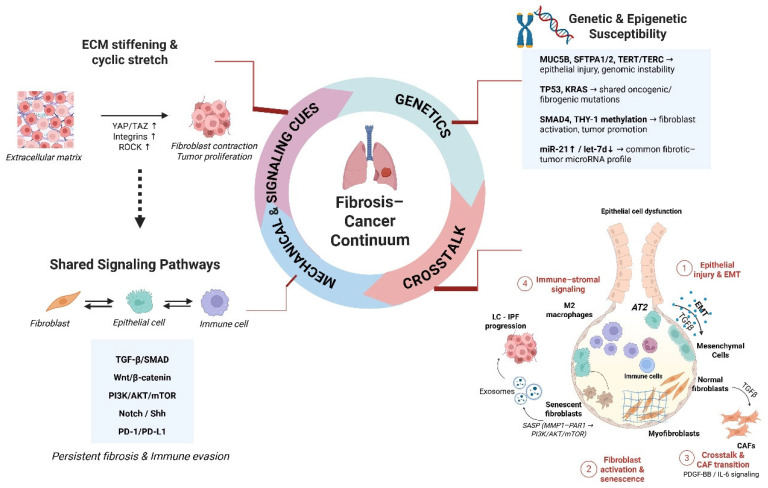
The fibrosis–cancer continuum illustrating shared pathogenic mechanisms between IPF and lung cancer. Genetic and epigenetic susceptibilities, mechanical cues, and common signaling pathways promote epithelial injury, fibroblast activation, and immune–stromal crosstalk, driving persistent fibrosis and tumor progression.

To this end, highlighting the pathogenic hallmarks of pulmonary fibrosis and lung cancer (Figure 1) could provide a better understanding of clinical and molecular commonalities and improve our knowledge on potential common therapeutic targets, fueling clinical trials and management algorithms that could improve clinical outcomes. This review provides an integrated perspective on the IPF–lung cancer relationship by combining genetic/epigenetic alterations, immune–stromal interactions, mechanical cues and shared therapeutic targets into a unified conceptual framework. A targeted literature search was conducted in PubMed, Scopus and Web of Science (2000–2025) using terms related to IPF, lung cancer, common pathogenetic mechanisms and emerging treatments. We prioritized peer-reviewed original studies, meta-analyses and high-quality reviews, while case reports were excluded unless mechanistically informative. When conflicting evidence arose, preference was given to larger studies or meta-analyses, and discrepancies were reported when relevant.

## 2. Histological Subtypes and Parenchymal Distribution of Idiopathic Pulmonary Fibrosis and Lung Cancer

In the general population, the most frequent histological subtype of lung cancer is adenocarcinoma [28]. However, in IPF patients, the histologic predominance of lung cancer has been controversial over the past few years. Most recent studies have shown that squamous cell carcinoma represents the most encountered subtype, followed by adenocarcinoma in IPF [4,12]. In addition, some random cases of small cell lung cancer and large cell lung cancer have been reported [7,12].

Recent studies investigating the distribution of lung cancer in IPF patients showed that carcinomas were localized in the peripheral areas of the lung, i.e., in the lower lobes, usually in the border between non-fibrotic areas and honeycombing [29,30], whereas in non-IPF patients, lung cancer is usually developed in the upper lobes. Fueled by epidemiological research showing a strong relationship between IPF and lung cancer, several pathogenic studies have developed the phenomenon of “scar-cinoma” to describe the link between lung scaring and cancer development. This concept reflects a distinct histotype of lung cancer with unique characteristics related to (a) histology type: squamous metaplasia, (b) location: the periphery of the lung within fibrotic and honeycombing regions and (c) tumors arising from excessively proliferating preneoplastic areas in the bronchiolar epithelium, located within honeycomb cysts [31]. Similarly, a linear association between hepatocellular carcinoma and liver cirrhosis has been reported [32]. These characteristic spatial patterns likely reflect selective pressures within the fibrotic microenvironment, where ECM remodeling, epithelial plasticity and immune dysregulation create a permissive niche for malignant transformation at fibrotic borders.

## 3. Common Pathogenetic Mechanisms Between Idiopathic Pulmonary Fibrosis and Lung Cancer

### 3.1. Genetics

At present, there is a growing interest in the genetic landscape of both clinical entities, revealing significant overlaps that underscore the common vulnerabilities in these diseases. Large genome-wide association studies have identified various genetic variants that contribute to nearly 30% of IPF cases. These genetic factors, combined with environmental risks, significantly influence the development of both sporadic and familial forms of IPF [33]. Among them, the single nucleotide polymorphism rs35705950 in the promoter region of the mucin 5B (MUC5B) gene has been shown to have the strongest association with both sporadic and familial types of IPF. Under physiological conditions, MUC5B contributes to mucociliary clearance and epithelial homeostasis. In contrast, its overexpression leads to impaired mucus transport, chronic epithelial injury, and dysregulated repair [34,35]. Importantly, this persistent injury is thought to create a permissive niche for DNA damage, metaplastic remodeling, and clonal evolution, thereby increasing susceptibility to malignant transformation [36].

Furthermore, current data suggest that some germline alterations that predispose individuals to IPF represent risk factors for lung carcinoma. Specifically, two heterozygous missense mutations in the SFTPA2 gene (single-base substitutions at codons 198 and 231) and one in the SFTPA1 gene (p. Trp211Arg) have been discovered in families with both lung carcinoma and IPF [37]. These mutations are expected to alter the structure of surfactant protein A (SP-A) and hinder its secretion, resulting in protein instability, endoplasmic reticulum stress in alveolar type II (ATII) cells and apoptosis. Endoplasmic reticulum stress contributes to oxidative damage and genomic instability, two hallmarks of epithelial carcinogenesis, and may promote malignant transformation of chronically injured epithelial lineages [38,39,40,41].

In addition, two genetic alterations in the compounds of the telomerase complex, TERT (telomerase reverse transcriptase) and TERC (telomerase RNA component), have been described in both conditions, IPF and lung cancer. Critically shortened telomeres, following progressive cell divisions, can provoke DNA damage responses resulting in genomic instability and premature cellular aging, thereby contributing to scarring and fibrotic remodeling in IPF. Consistent with this, peripheral blood leukocyte telomere length has been identified as a strong predictor of mortality in IPF [42,43,44]. In lung cancer, however, telomerase activation represents a central mechanism through which malignant cells acquire replicative immortality. Reactivation or overexpression of TERT allows cells with genomic instability to sustain unlimited proliferation, supporting clonal expansion and tumor progression irrespective of traditional risk factors [37]. Moreover, mutation carriers of the TERT rs2736100 variant show increased susceptibility to lung adenocarcinoma, and other genetic variants in TERC, OFBC1 and RTEL1 have been identified as heritable risk factors for lung cancer development [45,46]. Indeed, an addition at chromosomal region 5p15.33 in TERT represents one of the most common genetic alterations in the early stages of NSCLC [47]. Taken together, these observations suggest that telomere dysfunction in IPF may not only accelerate fibrotic remodeling but also promote selection of aberrant epithelial lineages capable of malignant transformation. This shared telomere biology may therefore represent a mechanistic bridge between chronic epithelial injury and oncogenesis in fibrotic lungs.

A recent meta-analysis of gene-expression datasets in IPF identified a set of differentially expressed genes (TNC, CDH2, SERPINA1, IL6, CYR61, SPP1) that are implicated not only in fibrotic remodeling but also in tumor invasion and progression, suggesting convergence between fibrogenic and oncogenic signaling programs [48]. Beyond transcriptional changes, somatic alterations in TP53, p16, KRAS and FHIT have been detected in both IPF tissues and lung tumors, supporting the concept that chronic epithelial injury and defective DNA repair promote genomic instability and clonal selection [41,49,50]. A point alteration in codon 12 of KRAS was not detected in lung carcinoma but in patients with concurrent lung cancer and IPF, indicating that the fibrotic microenvironment may shape distinct evolutionary pathways of carcinogenesis [49,51]. Moreover, biomarker studies lend further support. The PROFILE study reported a serum biomarker signature enhanced with cancer-related genes CA125 and CA19-9, indicative of worse clinical outcomes in IPF patients [52], while Allen et al. reported somatic alterations, among which TP53 and BRAF were significantly mutated in IPF-lung cancer [53].

### 3.2. Epigenetics

IPF and lung cancer present with common environmental risk factors such as smoking and occupational exposures, but also exhibit convergent epigenetic alterations that influence cellular aging, senescence and malignant transformation. DNA methylation patterns, histone modification enzymes and aberrant regulation of non-coding RNAs have a cardinal role as epigenetic stamps. Hypermethylation of the SMAD4 promoter, for example, results in reduced SMAD4 expression in patients with concomitant IPF and lung cancer, contrasting with its overexpression in isolated lung cancer, and suggesting context-dependent regulation of TGF-β signaling and epithelial plasticity [54]. Similarly, THY-1 hypermethylation contributes to fibroblast-to-myofibroblast differentiation in IPF and is linked to metastatic behavior in lung cancer cells [55,56]. Conversely, the O-6-methylguanine DNA methyltransferase (MGMT) gene exhibits hypermethylation in lung carcinomas and hypomethylation in fibroblasts of IPF lungs, reflecting divergent effects of DNA repair dysregulation in fibrotic versus neoplastic settings [41,57].

In addition, histone modification enzymes such as histone demethylase and deacetylase inhibitors play crucial roles in regulating gene expression in both lung cancer and pulmonary fibrosis. Dysregulation of these enzymes, i.e., upregulation of CDKN1A/ p21waf1/cip1 and Fas, can lead to aberrant gene expression patterns that promote oncogenesis and fibrosis, making them potential targets for therapeutic intervention [4,58,59]. A plethora of microRNAs present abnormal expression in both pulmonary fibrosis and lung cancer. For example, miR-21 has been found highly upregulated in the serum of IPF patients, and its overexpression represents a negative prognostic factor for overall survival in NSCLC [60,61]. By contrast, miR-29 and let-7d were mostly downregulated in both conditions, influencing ECM accumulation, epithelial differentiation and malignant progression [62,63]. These data indicate that a unique epigenomic profiling may drive both maladaptive repair and tumorigenesis in fibrotic lungs, highlighting a potential epigenomic basis for the fibrotic–cancer continuum. These convergent genetic and epigenetic alterations illustrate how persistent epithelial injury, genomic instability and maladaptive repair programs may jointly drive fibrogenesis and malignant transformation, reinforcing the concept of a shared fibrotic–cancer continuum (Table 1).

### 3.3. Principal Fibrogenic Molecules and Signaling Pathways

Fibrogenic-associated signal transduction pathways represent a major part of the pulmonary fibrosis–lung cancer continuum. TGFβ1 is the master regulator of lung remodeling and is often overexpressed in both diseases. In IPF, it drives the apoptosis of alveolar epithelial cells, chemoattraction of monocytes and macrophages, fibroblast activation, myofibroblast differentiation, and extracellular matrix (ECM) deposition, leading to fibrotic lesions. It is also known to elicit epithelial–mesenchymal transition (EMT) [64,65]. In the context of lung cancer, TGFβ triggers disease progression and tumor metastasis, inducing EMT and suppressing immune surveillance. It also plays an important role in tumor stroma development by activating cancer-associated fibroblasts, linking maladaptive tissue repair to malignant progression [66].

Tyrosine kinase receptor ligands represent a key regulatory pathway with aberrant expression in lung cancer and pulmonary fibrosis. For example, PDGF stimulates fibroblast proliferation and ECM accumulation in IPF, but also enhances tumor growth, angiogenesis and lymphangiogenesis in lung carcinoma, suggesting that PDGF-driven fibroblast activation may create a supportive stromal niche for invasion [41]. In contrast, CTGF, although pro-fibrotic in IPF, is suppressed in NSCLC, where it can limit invasion and metastasis [67]. These context-dependent roles highlight that molecules driving fibrosis can function as tumor promoters or suppressors depending on cellular lineage, microenvironmental cues, and oncogenic state.

Paracrine communication via extracellular vesicles further reinforces the fibrotic–cancer continuum. IPF fibroblast-derived exosomes are enriched in MMPs, integrins and annexins, and transfer bioactive cargo to epithelial and immune cells, promoting EMT, tissue remodeling and tumor-permissive signaling [68]. Dysregulation of connexins such as Cx43 further disrupts epithelial–mesenchymal communication and has been associated with both fibroblast persistence and tumor progression [4].

LPA-related signaling represents another point of convergence. Elevated LPA drives fibroblast activation and vascular leakage in experimental fibrosis [69], while the autotaxin/LPA axis has recently been implicated in lung cancer proliferation and metastasis [70], suggesting a shared lipid-mediated pathway supporting fibrosis and tumorigenesis. Similarly, chronic inflammation induces continuous epithelial injury and aberrant repair in both diseases. IL-13 promotes fibroblast differentiation via TGF-β pathways [41,71] and is associated with poor clinical outcomes in NSCLC, providing a mechanistic link between inflammatory remodeling and cancer aggressiveness [72,73].

Major signal transduction pathways are implicated in the pathogenetic mechanisms of IPF and lung cancer. Aberrant activation of the Wnt/b-catenin pathway is a critical regulator of cancer metaplasia, but also regulates expression of cyclin-D1 and matrix metalloproteinase-7 (MMP-7), and thus leading to irreversible scarring and remodeling of lung tissue [74,75]. Moreover, the phosphoinositide 3-kinase (PI3K)/protein kinase B (AKT) signaling pathway has been correlated with fibroblast proliferation in IPF as well as with high-grade tumors and advanced disease [76,77]. It is more commonly altered in squamous cell lung carcinoma than in adenocarcinoma [78]. Fibroblast-derived osteoprotegerin (OPG) has recently been identified as another shared mediator between fibrosis and lung cancer, as OPG-mediated ERK activation drives fibroblast activation in IPF and enhances tumor cell proliferation in NSCLC [79,80]. Abnormal mechanical forces in the fibrotic lung microenvironment, such as ECM stiffening and cyclic stretch, activate mechanosensitive pathways (e.g., YAP/TAZ, integrins, ROCK) that enhance both fibroblast activation and cancer cell proliferation, underscoring the mechanical–molecular bridge in the fibrosis–cancer continuum [80].

Developmental morphogens further contribute to shared programs. Sonic Hedgehog signaling is upregulated in epithelial cells near honeycombing, promoting EMT and fibroblast resistance to apoptosis [81], while in lung cancer it drives cancer stemness, progression and treatment resistance [82]. Moreover, activation of the Notch signaling pathway promotes fibrotic responses by inducing α-SMA expression in fibroblasts and EMT in alveolar epithelial cells [83] and supports proliferation and survival of cancer cells in NSCLC, highlighting a common epithelial plasticity axis [84]. Therefore, targeting the Notch signaling pathway holds potential for therapeutic interventions in both lung cancer and IPF, although the complexity and context-dependent nature of this pathway need careful consideration of treatment strategies.

**Figure 2:**
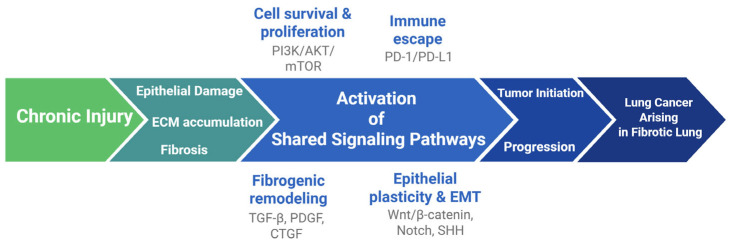
Recurrent epithelial injury and fibrosis activate signaling pathways (TGF-β, PDGF, CTGF, Wnt/β-catenin, PI3K/AKT/mTOR, SHH, Notch, PD-1/PD-L1) that drive EMT, proliferation, stemness and immune evasion, facilitating tumor initiation and progression within the fibrotic lung.

Finally, immune checkpoint dysregulation provides an additional point of convergence. The programmed death ligand-1/ programmed cell death 1 (PD-L1/PD-1) axis, central to immune escape in NSCLC, is elevated in mediastinal lymph nodes of IPF patients and experimental fibrosis models. Low-dose PD-1 blockade attenuated fibrosis in bleomycin models suggests that immune checkpoint inhibition may exert both anti-tumor and anti-fibrotic effects. Clinical trials endotyping patients based on the profile of mediastinal lymphadenopathy and implementing targeted therapies based on mediastinal node PD-1 expression are highly anticipated [85,86]. These key pathways are summarized in Figure 2, highlighting convergent molecular programs underlying fibrogenesis and malignant progression.

### 3.4. Epithelial–Mesenchymal Crosstalk

EMT is a procedure by which epithelial cells lose their polarity and cell-to-cell adhesion properties, obtaining mesenchymal traits that enhance their migratory and invasive capacity. Specifically, lung injury induces the activation of myofibroblasts to proliferate and secrete ECM components such as collagen type I and α-smooth muscle actin (α-SMA). Studies have shown that several cell types contribute to the myofibroblast pool within the fibrotic niche, such as resident fibroblasts, AECIIs, circulating fibrocyte endothelial cells, adipocytes and pericytes. In particular, injured alveolar epithelial type II cells have the potential to acquire mesenchymal characteristics and thus undergo phenotypic changes. These mesenchymal-like cells can serve as the pool of activated fibroblasts and myofibroblasts in the fibrotic foci, inducing the excessive production of ECM and lung scaring [41,87,88]. Furthermore, there is an emerging theory that circulating fibrocytes, originating from the bone marrow, have the potential to act as myofibroblast precursors following TGFβ and endothelin 1 stimulation, and contribute to lung fibrosis [41,89,90], and endothelial to mesenchymal transition in pulmonary fibrosis, indicative of poor prognosis [91].

Similar EMT-driven programs are observed in lung cancer, where cancer-associated fibroblasts (CAFs) form a supportive tumor niche that sustains proliferation, invasion, and stemness. Circulating fibrocytes and EMT-derived epithelial cells can also contribute to CAF pools during tumor initiation and metastasis [92]. CAF-derived cytokines such as CLCF1 and IL-6 exert paracrine pro-tumorigenic effects [93], while CXCL-12/CXCR4 and IGF-II/IGF-R1 signaling further potentiate stromal–tumor crosstalk [94,95]. Immune–stromal interactions also reinforce EMT in IPF-associated lung cancer: M2 macrophages and β-catenin-dependent CXCL6 signaling promote fibroblast–cancer communication and invasive behavior [96,97]. Likewise, in the tumor microenvironment, vascular pericytes constitute an important compound as they have been correlated with tumor vasculature. More specifically, PDGF-BB seems to promote pericyte–fibroblast transition (PFT) suggesting a role in tumor invasion and metastasis, and thus targeting PFT as a possible therapeutic option [98]. Finally, the inheritance of mesenchymal phenotype by tumor epithelial cells leading to loss of cell-to-cell adhesion and increased resistance to apoptosis is responsible for metastasizing [99]. Targeting EMT and stromal remodeling therefore represents a promising therapeutic avenue in IPF-associated lung cancer.

### 3.5. Mechanical Stress and the Fibrotic–Cancer Mechano-Nexus

A defining feature of IPF is the progressive stiffening of the lung parenchyma due to excessive extracellular matrix deposition and abnormal collagen cross-linking. This mechanical alteration profoundly influences cellular behavior. Fibroblasts and myofibroblasts exposed to increased matrix stiffness exhibit enhanced contractility, sustained activation, and reduced susceptibility to apoptosis, creating a feed-forward loop that reinforces fibrotic remodeling [100]. Key mechanotransduction pathways, mediated by integrins, focal adhesion kinase (FAK), Rho/ROCK signaling and downstream activation of YAP/TAZ, translate these mechanical cues into transcriptional programs that maintain the fibrotic phenotype [101,102].

The same mechano-niches that perpetuate fibrosis also create a permissive environment for malignant transformation and tumor progression. In cancer biology, increased ECM stiffness has been shown to enhance proliferation, invasion, EMT, and resistance to therapy through integrin/FAK signaling and YAP/TAZ-driven transcriptional programs. Lung cancer arising in fibrotic lungs thus develops in a biomechanically abnormal scaffold, where stiff ECM and activated CAFs support tumor cell survival, promote invasive growth along fibrotic tracts, and facilitate immune escape, partly via YAP-dependent regulation of immune checkpoints such as PD-L1 [80,103,104]. Recent conceptual work has proposed that aberrant mechanical forces in IPF may represent a “missing link” between chronic fibrosis and lung cancer initiation, integrating structural distortion of the lung, persistent epithelial injury and oncogenic reprogramming within shared mechanotransduction pathways [105].

### 3.6. Senescence

Senescence serves as a crucial biological mechanism to prevent the proliferation of damaged or dysfunctional cells, thus acting as a tumor-suppressive response and playing a role in tissue homeostasis. In IPF and lung cancer, however, senescence acquires a dual and context-dependent role. In the fibrotic lung, senescent fibroblasts generate a complex senescence-associated secretory phenotype (SASP) enriched in profibrotic cytokines, matrix-remodeling enzymes and growth factors, which promotes fibroblast activation, ECM deposition and resistance to apoptosis [106]. Although senescence can restrain tumor growth by impairing replicative capacity, accumulating evidence shows that SASP produced by senescent fibroblasts may also enhance malignant progression by creating a pro-inflammatory and proteolytic microenvironment [41,107].

Recent studies provide mechanistic insight into this process. Senescent fibroblasts from IPF lungs have been shown to secrete exosomal MMP1, which activates PAR1 signaling in NSCLC cells and subsequently triggers PI3K/AKT/mTOR pathways, promoting EMT features and invasive behavior. Pharmacologic inhibition of MMP1 or PAR1 attenuates these effects, demonstrating a direct contribution of the senescent fibrotic stroma to tumor progression [108]. Furthermore, EVs considered a significant component of the SASP may mediate paracrine signaling in IPF. Notably, fibroblast-derived EVs carrying SFRP1 can activate Wnt/β-catenin signaling in alveolar type II cells, while miR-19a within EVs regulates the ZMYND11/c-Myc axis in lung cancer cells, implicating a direct EV-mediated link between the fibrotic microenvironment and tumorigenesis [80,109].

In addition, immune–stromal elements amplify these senescence-driven effects. M2-polarized macrophages and CAF-like activated fibroblasts reinforce SASP-mediated inflammation, remodel the matrix, and support epithelial vulnerability to transformation. Collectively, these interactions establish a permissive microenvironment in which chronic senescence promotes both persistent fibrosis and the early steps of carcinogenesis, highlighting senolytic pathways as potential dual anti-fibrotic and anti-tumor therapeutic targets [110].

## 4. Molecular Profiling and Clinical Implications

The molecular profiling of IPF-related lung cancer reveals patterns that differ from those observed in sporadic disease, reflecting the distinct biology of carcinogenesis within fibrotic tissue. Several studies have identified recurrent alterations in genes involved in cell-cycle regulation, genomic stability, and oxidative responses. Specifically, SETD2 and NFE2L2 mutations, as well as MYC amplification, are more frequently observed in patients with squamous NSCLC arising in the context of IPF and have been associated with poorer prognosis [111]. KRAS mutations, including the G12C variant, have been detected in the metaplastic bronchiolar epithelium in UIP lungs, supporting the concept that chronic epithelial injury and metaplasia may constitute a precursor state for malignant transformation [112]. In contrast, EGFR mutations remain uncommon in IPF-associated lung cancer, as demonstrated by studies from Guyard et al. and Honda et al., indicating a molecular landscape distinct from that of sporadic adenocarcinoma [20,113]. Moreover, BRAF and TP53 mutations appear to be enriched in IPF-related cancers, further underscoring shared mechanisms of genomic instability and dysregulated epithelial repair [114,115].

Taken together, these molecular features have direct therapeutic implications. Lung adenocarcinomas arising within fibrotic lungs often exhibit EGFR–wild type and KRAS-mutated profiles, partly due to the increased prevalence of invasive mucinous and related mucinous variants in interstitial pneumonia, histologic subtypes that characteristically harbor KRAS mutations. This mechanistic context highlights the potential relevance of KRAS-targeted therapy in IPF-associated lung cancer. Recent real-world data demonstrate that KRAS G12C inhibitors show meaningful anti-tumor activity with acceptable tolerability in NSCLC patients with comorbid interstitial pneumonia, supporting their consideration in carefully selected cases [115,116].

Beyond oncogene-directed approaches, immunotherapy has also attracted interest in this population. Studies have shown elevated PD-1 expression and altered PD-L1 profiles in mediastinal lymph nodes of patients with IPF, while preclinical models demonstrate that PD-1 blockade may attenuate fibrosis in addition to enhancing anti-tumor immunity [85]. Recent findings in progressive fibrotic ILDs further suggest shared adaptive immune signatures, including germinal center B-cell and T-follicular helper cell activation, that may represent targetable pathways across both disease processes [117]. Although TKIs and ICIs thus offer a promising therapeutic axis with potential anti-fibrotic effects, their use in patients with concurrent IPF and lung cancer requires careful patient selection due to the risk of treatment-related exacerbation of underlying ILD.

## 5. Future Perspectives

Despite the mechanistic links between pulmonary fibrosis and lung cancer, evidence-based guidelines about the therapeutic management of the concurrence of the two entities remain a matter of debate. The Japanese Respiratory Society statement represents the most comprehensive attempt so far to systematize the treatment of lung cancer with comorbid interstitial pneumonia; however, its recommendations are largely based on retrospective data and expert consensus, highlighting the urgent need for prospective validation and international harmonization of management algorithms [118].

In this context, a pragmatic clinical framework may help guide decision-making until formal guidelines become available. Assessing ILD severity, pulmonary function and HRCT pattern is essential, as these parameters largely determine the risk of treatment-related acute exacerbation and influence the diagnostic approach. Invasive diagnostic procedures, particularly transthoracic biopsy and thoracic surgery, carry a considerable risk of postoperative acute exacerbation, especially in patients with a UIP pattern and limited functional reserve, and should be considered with caution. High inspired oxygen fractions, lobar or bi-lobar surgical approaches leading to prolonged mechanical ventilation and thoracic surgical stress, as well as fluid imbalance, are recognized triggers of acute exacerbation in IPF and should be minimized whenever possible through lung-protective strategies and careful peri-operative management. Less invasive diagnostic approaches, close radiologic surveillance and risk-adapted decision-making are often preferable in this high-risk group. Multidisciplinary discussion involving ILD expert pulmonologists, oncologists, thoracic radiologists, anesthesiologists and thoracic surgeons remains central for minimal peri-operative risks and optimal therapeutic benefits for this fragile patient subpopulation.

Therapeutic decision-making remains challenging, as several conventional lung cancer treatments may precipitate ILD progression or trigger acute exacerbations. Nevertheless, recent studies provide encouraging evidence that platinum-based chemotherapy and immune checkpoint inhibitors can be feasible in appropriately selected patients with fibrotic ILD and lung cancer [6,119]. A nationwide population-based study by Karayama et al. demonstrated improved survival with ICIs compared with cytotoxic chemotherapy in NSCLC patients with ILD, despite a higher incidence of treatment-related pneumonitis, supporting their judicious use in this high-risk population [120].

Accumulating data also suggest that anti-fibrotic therapy should generally be maintained following a diagnosis of lung cancer in patients with IPF. A multicenter observational analysis showed that anti-fibrotic treatment was associated with lower incidence and reduced mortality from lung cancer among patients with IPF, indicating a potential protective effect against tumor development [121]. More importantly, a recent systematic review and meta-analysis by Srivali et al. [122] documented peri-operative beneficial effects in patients with IPF undergoing lung cancer surgery. In particular, the analysis revealed that peri-operative anti-fibrotic therapy (pirfenidone) at different dose and time regimens achieved (a) 69% reduction in AE-IPF (RR: 0.31, 95% CI: 0.13–0.70), (b) 81% reduction in 90-day mortality (RR: 0.19, 95% CI: 0.07–0.52) (c) shorter hospitalization days (5 vs. 7 days, *p* = 0.029), as well as reduced leak incidence (*p* = 0.021), and (d) an acceptable safety profile as indicated by only mild side effects reported (nausea, photosensitivity) with no severe adverse events or treatment discontinuations [122]. Notably, nintedanib’s dual anti-fibrotic and anti-cancer activity has been demonstrated in several settings [123]. To this end, the first randomized controlled study (J-SONIC) showed that nintedanib combined with carboplatin and nab-paclitaxel improved outcomes in patients with IPF and advanced NSCLC of non-squamous histology [124]. There is also abundant evidence showing the efficacy of nintedanib combined with docetaxel as a second-line option for patients with advanced-stage NSCLC, especially with adenocarcinoma [125]. The LUME-Lung 2 study has demonstrated that nintedanib along with pemetrexed significantly prolongs progression-free survival in pretreated advanced NSCLC [126]. Beyond NSCLC, the phase II NEXT-SHIP trial demonstrated manageable safety and encouraging activity of carboplatin–etoposide plus nintedanib in small cell lung cancer with comorbid IPF, a group at particularly high risk for chemotherapy-induced exacerbations [127]. Towards this direction, short-term peri-operative anti-fibrotic treatment has been associated with a reduced risk of postoperative acute exacerbation and improved short-term survival, supporting its role as a risk mitigation strategy in operable disease, although optimal timing and duration remain to be defined [128]. Preclinical data further point to potential synergy between anti-fibrotics and cytotoxic agents, with enhanced apoptosis in cancer-associated fibroblasts and tumor cells, providing mechanistic support for combination regimens [129]. Prognostically, patients with concomitant IPF and lung cancer have significantly worse survival than patients with either condition alone, driven by both tumor aggressiveness and ILD-related mortality, as well as treatment limitations imposed by reduced physiological reserve.

Although no formal screening recommendations exist, emerging evidence suggests that patients with fibrotic ILD—particularly those with progressive UIP—may benefit from closer radiologic surveillance for early detection of lung cancer, given their elevated incidence and poorer surgical candidacy. Progress in this field is hindered by the systematic exclusion of patients with fibrotic ILD from lung cancer clinical trials, resulting in evidence gaps and limited generalizability. Future research should prioritize dedicated studies enrolling patients with both conditions, prospective registries capturing real-world outcomes, and clinically relevant stratification tools that incorporate molecular, radiologic and functional risk markers. Taken together, these observations emphasize that the coexistence of IPF and lung cancer is not only a biological continuum but also a clinical entity defined by increased procedural risk, limited physiological reserve and narrow therapeutic margins. The development of standardized diagnostic workflows, peri-operative protocols and integrated treatment algorithms will be essential to improve outcomes in this high-risk and clinically vulnerable population (Table 2 and Table 3).

## 6. Conclusions

IPF represents an independent risk factor for lung cancer development. This review aims to analyze common potential pathogenetic mechanisms including genetic predisposition, cellular responses and molecular pathways between both entities. Currently, there is no consensus regarding the therapeutics of concomitant IPF and lung cancer. The potential synergistic effects of anti-fibrotic and anti-cancer compounds open novel therapeutic avenues for the treatment of diseases with complex disease pathophysiology and poor clinical outcomes. The European Respiratory Society has recently launched a task force to standardize management approaches for increased awareness, prevention and treatment of these diseases, which holds promising results. There is an urgent need for clinical trials that could prospectively evaluate the effectiveness of currently approved anti-fibrotic drugs in patients with both IPF and lung cancer to improve patients’ survival and quality of life.

## Figures and Tables

**Table 1:** Principal genetic and epigenetic mechanisms between IPF and lung cancer.

Molecule	Role in IPF	Role in Lung Cancer
Genetic		
MUC5B (rs35705950)	Impaired mucus clearance, chronic epithelial damage	Persistent epithelial stress facilitating carcinogenesis
SFTPA1/SFTPA2 mutations	Disrupted surfactant, ATII apoptosis, fibrosis	Loss of homeostasis, oncogenic transformation
TERT/TERC	Shortened telomeres, DNA damage, premature cell aging, scarring	Telomerase reactivation enabling replicative immortality
TP53, KRAS, p21, p16, BRAF	Changes in DNA repair genes, fibroblast survival	Early driver events, clonal expansion, tumor progression
Epigenetic		
SMAD4 hypermethylation	Reduced SMAD4 expression, dysregulated TGF-β signaling, fibrogenesis	Loss of tumor suppression, enhanced EMT and invasion
THY-1 hypomethylation	Differentiation of fibroblasts into myofibroblasts	CAF-like phenotype, metastatic potential
MGMT	Hypomethylation in fibroblasts of IPF, altered DNA repair	Hypermethylation leading to impaired repair and oncogenesis
miR-21 abnormal expression	Fibroblast activation, profibrotic signaling	Aggressive tumor phenotype, immune evasion
miR-29 (downregulation)	ECM accumulation, fibroblast activation	Tumor invasiveness, EMT, Metastasis
let-7d (downregulation)	Increase TGF-β signaling and fibroblast proliferation	Poor prognosis, oncogene activation

Abbreviations: ATII: alveolar type II epithelial cells; BRAF: v-Raf murine sarcoma viral oncogene homolog B; CAF: cancer-associated fibroblast; DNA: deoxyribonucleic acid; ECM: extracellular matrix; EMT: epithelial–mesenchymal transition; KRAS: Kirsten rat sarcoma viral oncogene homolog; MGMT: O6-methylguanine-DNA methyltransferase; miR/miRNA: microRNA; p16: cyclin-dependent kinase inhibitor 2A; p21: cyclin-dependent kinase inhibitor 1A; SFTPA1/SFTPA2: surfactant protein A1/A2; SMAD4: SMAD family member 4; TGF-β: transforming growth factor-beta; TERT/TERC: telomerase reverse transcriptase/telomerase RNA component; THY-1: thymocyte differentiation antigen 1; TP53: tumor protein p53.

**Table 2:** Core diagnostic elements influencing evaluation and risk stratification in patients with IPF and suspected lung cancer.

Clinical Domain	Key Considerations
ILD Severity	FVC, DLCO, HRCT pattern, risk of AE-ILD
Radiological Evaluation	Morphology growth, risk-adapted surveillance
Procedural Risk	Increased risk of AE-ILD with biopsy/surgery, especially in UIP cases
Physiological Reserve	Limited gas exchange capacity may restrict invasive diagnostics
MDD	Essential to balance oncologic yield and pulmonary risk

Abbreviations: AE-ILD: acute exacerbation of interstitial lung disease; DLCO: diffusing capacity of the lung for carbon monoxide; FVC: forced vital capacity; HRCT: high-resolution computed tomography; ILD: interstitial lung disease; MDD: multidisciplinary discussion; UIP: usual interstitial pneumonia.

**Table 3:** Summary of key therapeutic principles balancing oncologic benefit and pulmonary safety in patients with IPF and lung cancer.

Treatment Domain	Key Considerations
Anti-fibrotic therapy	Continue when feasible; may reduce cancer incidence/mortality
Platinum-based chemotherapy	Feasible in selected patients; risk of AE-ILD
Immune checkpoint inhibitors	Survival benefit but higher pneumonitis risk; careful monitoring
Targeted therapies	Nintedanib with docetaxel/pemetrexed improves outcomes
Surgical management	Increased peri-operative AE risk
Peri-operative precaution measures	Peri-operative use of pirfenidone, apply protective ventilation strategies, ensure negative fluid balance, minimize FiO_2_, consider lung volume preservation surgical approaches to mitigate AE risk

## Data Availability

No new data were created or analyzed in this study.
